# The polymorphisms of *ANXA6* influence head and neck cancer susceptibility in the Chinese Han population

**DOI:** 10.3389/fonc.2023.1100781

**Published:** 2023-03-14

**Authors:** Weihong Xiong, Zhumei Li, Xiangfa Zeng, Jun Cui, Zhiming Cheng, Xiaoying Yang, Yipeng Ding

**Affiliations:** ^1^ Department of Otorhinolaryngology Head and Neck Surgery, People’s Hospital of Wanning, Wanning, Hainan, China; ^2^ Department of General Practice, Hainan General Hospital, Hainan Affiliated Hospital of Hainan Medical University, Haikou, Hainan, China

**Keywords:** head and neck cancer, *ANXA6*, single nucleotide polymorphism, case-control study, Chinese Han population

## Abstract

**Background:**

Head and neck cancer (HNC) is the sixth most common malignant tumor worldwide and imposes a serious economic burden on society and individuals. Annexin has been implicated in multiple functions which are essential in HNC development, including cell proliferation, apoptosis, metastasis, and invasion. This study focused on the linkage between *ANXA6* variants and HNC susceptibility in Chinese people.

**Methods:**

Eight SNPs in *ANXA6* from 139 HNC patients and 135 healthy controls were genotyped by the Agena MassARRAY platform. The correlation of SNPs with HNC susceptibility was evaluated using odds ratio and 95% confidence interval calculated by logistic regression using PLINK 1.9.

**Results:**

Overall analysis results demonstrated that rs4958897 was correlated with an increased HNC risk (allele: OR = 1.41, *p* = 0.049; dominant: OR = 1.69, *p* = 0.039), while rs11960458 was correlated with reduced HNC risk (OR = 0.54, *p* = 0.030). In age ≤ 53, rs4958897 was related to reduce HNC risk. In males, rs11960458 (OR = 0.50, *p* = 0.040) and rs13185706 (OR = 0.48, *p* = 0.043) were protective factors for HNC, but rs4346760 was a risk factor for HNC. Moreover, rs4346760, rs4958897, and rs3762993 were also correlated with increased nasopharyngeal carcinoma risk.

**Conclusions:**

Our findings suggest that *ANXA6* polymorphisms are linked to the susceptibility to HNC in the Chinese Han population, indicating that *ANXA6* may serve as a potential biomarker for HNC prognosis and diagnosis.

## Introduction

Head and neck cancer (HNC) is the seventh most common malignant tumor worldwide, which is a squamous cell carcinoma that occurs in the lip, oral cavity, pharynx, and larynx (1). The global cancer burden using the GLOBOCAN 2020 estimation of cancer incidence and mortality produced by the International Agency for Research on Cancer (IARC) is estimated to be 931,931 new HNC cases and 467,125 HNC deaths in 2020. There will be approximately 148,344 new HNC cases and 78,554 HNC deaths in China in 2022 (2). The treatment regimens for HNC are complicated and bring a heavy burden to patients, often affecting their speech, swallowing, and respiratory functions (3). Therefore, it is necessary and urgent to explore the pathological mechanism of HNC.

HNC is a multifactorial disease that may be caused by complex factors, including environmental and genetic factors. Previous studies have indicated that tobacco smoking, excessive alcohol consumption and human papillomavirus (HPV) infection could contribute to the occurrence and development of HNC (3–5). In recent years, a study has demonstrated that individuals with a family history of HNC have an increased risk of HNC approximately two to three-fold (6). However, only a small proportion of individuals will eventually develop HNC. Genetic mutations such as single nucleotide polymorphisms (SNPs) may potentially alter the susceptibility of an individual to HNC. Several studies have identified that genetic polymorphisms of *TCF19* (7), *CYP2B6*, *HSD17B12* (8), *GSTM1*, and *GSTT1* (9) are associated with HNC risk. Taken together, these findings reveal that genetic mutations play an important role in tumorigenesis and increase the risk of HNC.

Annexinis a kind of calcium ion-dependent phospholipid binding protein. A great deal of literature has reported that annexin plays a key role in multiple functions essential in cancer, including cell proliferation, apoptosis, chemosensitivity, metastasis, and invasion (10–13). Notably, the role of annexin in HNC development has attracted widespread attention. For example, Chen et al. have found that the overexpression of *ANXA2* is correlated with a poor prognosis of HNC (14). Salom et al. have shown that *ANXA9* and *ANXA10* are abnormally expressed in HNC tissues and are related to the grade of tumor differentiation (15). A study has indicated that *ANXA1* promotes nasopharyngeal carcinoma growth and metastasis *via* the binding and stabilization of EphA2 (16). *ANXA6* has been reported to be closely associated with a variety of tumors and be involved in cancer cell growth, motility, invasion, and adhesion (17). Xin Sun et al. have showed that *ANXA6* suppresses the tumorigenesis of cervical cancer through autophagy induction (18). *ANXA6* induces gemcitabine resistance by inhibiting ubiquitination and degradation of *EGFR* in triple-negative breast cancer (19). Polymorphisms in the *ANXA6* gene were significantly associated with the risk of osteonecrosis of the femoral head (ONFH) (20), systemic lupus erythematosus (21). However, there is a lack of data on *ANXA6* polymorphisms in the occurrence and development of HNC.

Therefore, this study was planned to explore whether *ANXA6* gene polymorphisms affect the susceptibility to HNC in the Chinese Han population. Eight SNPs in the *ANXA6* gene were screened to evaluate the linkage between *ANXA6* variants and HNC susceptibility from 139 patients with HNC and 135 healthy controls. Our results may provide new ideas for the diagnosis and treatment of HNC.

## Materials and methods

### Study population

In total, 274 individuals from People’s Hospital of Wanning were recruitedin this study, including 139 HNC patients and 135 healthy controls. All patients were histologically diagnosed with HNC by two pathologists. Patients who had received chemotherapy or radiotherapy and had a history or family history of cancer were excluded. The inclusion criteria for the control group were: individuals without a history of cancer or chronic diseases.

### SNP selection and genotyping

A total of eight SNPs (rs11960458, rs4958892, rs78243462, rs4346760, rs4958897, rs3762993, rs9324677, and rs13185706) were screened from the *ANXA6* gene and then genotyped using the Agena MassARRAY system (Agena, San Diego, CA, U.S.A.) as described previously (22, 23). These SNPs had a minor allele frequency (MAF) >5% in the Chinese Han Beijing (CHB) population from the 1000 Genomes Project. Total DNA was extracted from peripheral blood using a DNA Extraction Kit (GoldMag, Xi’an, China). The concentration and purity of DNA were measured by NanoDrop 2000 (Thermo Scientific, USA). Data management was conducted by Agena Typer 4.0 software.

### Statistical analysis

We utilized t-test and χ^2^ test to analyze differencesin age and gender between cases and controls. Hardy-Weinberg equilibrium (HWE) of the control group was evaluated by χ^2^ test. Besides, odds ratio (OR) and 95% confidence interval (CI) were used to assess the linkage between *ANXA6* variants and HNC risk under the five genetics models (allele, genotypes, dominant, recessive and additive model)via logistic regression analysis using PLINK 1.9. One SNP has two alleles (A/a), and there are three genotypes (AA, Aa and aa). If “a” is regarded as a risk allele, in the additive model, a frequency is counted as long as there is one “a” in the genotype, that is, when the genotype is AA, Aa, or aa, the frequency is 0, 1, or 2, respectively. In the dominant model, the frequency is calculated once as long as there is one “a” without taking into account the quantity of “a”, similar to the qualitative method, that is, when the genotype is AA, Aa, or aa, the frequency is 0, 1, or 1, respectively. In the recessive model, the frequency is calculated only if there are two “a”s, that is, when the genotype is AA, Aa, or aa, the frequency is 0, 0, or 1, respectively. Multi-factor dimensionality reduction (MDR) was used to assess the effect of potential SNP-SNP interactions on HNC risk. *P* < 0.05 was considered to be statistically significant.

## Results

### Study population

This study included 139 patients with HNC (98 men and 41 women) and 135 healthy controls (95 men and 40 women). The mean age of the control group was 53.00 ± 10.81 years, and that of the case group was 53.05 ± 12.76 years (**Table 1**). No significant differences were observed in age (*p* = 0.972) and gender stratification between the case and control groups (*p* = 0.380).

**Table 1:** Demographic characteristics of HNC cases and controls.

Variables	Cases	Controls	*p* value
Total	139	135	
Age (years, mean ± SD)	53.05 ± 12.76	53.00 ± 10.81	0.972^a^
> 53	72 (52%)	72 (53%)	
≤ 53	67 (48%)	63 (47%)	
Gender			0.380^b^
Male	98 (71%)	95 (70%)	
Female	41 (29%)	40 (30%)	
Types of HNC			
Nasopharynx	77 (55%)		
Larynx	43 (31%)		
Parotid gland	19 (14%)		

SD, standard deviation.

p^a^ values were calculated from student’s t test.

p^b^ values were calculated from χ^2^ test.

p < 0.05 indicates statistical significance.

### Association of *ANXA6* SNPs with HNC risk

The primary information on *ANXA6* SNPs is listed in **Table 2**, and all SNPs met HWE (*p* > 0.05). It was revealed that our study population was in a state of genetic balance, and the genotyping results were reliable, meeting the requirements of random sampling. This study results indicateed that the C allele of rs4958897 was correlated with an increased risk of HNC compared with the T allele (OR = 1.41, 95% CI = 1.00-1.98, *p* = 0.049). No correlation was observed between the other seven *ANXA6* SNPs and susceptibility to HNC (*p* > 0.05).

**Table 2:** Primary information of selected SNPs in *ANXA6*.

SNP-ID	Chr	Position	Role	Cases	Controls	Alleles	MAF		HWE	OR(95%CI)	*p*
						A/B	Case	Control	*p*		
rs11960458	5	151100959	3’-UTR	124/154	119/151	T/C	0.446	0.441	0.299	1.02 (0.73-1.43)	0.901
rs4958892	5	151103534	Intron	94/184	99/171	A/G	0.338	0.367	0.094	0.88 (0.62-1.25)	0.484
rs78243462	5	151111165	Intron	23/255	20/250	T/C	0.083	0.074	0.532	1.13 (0.60-2.10)	0.706
rs4346760	5	151113909	Intron	150/128	125/145	C/A	0.54	0.463	0.301	1.36 (0.97-1.90)	0.073
rs4958897	5	151120172	Intron	127/151	101/169	C/T	0.457	0.374	0.142	1.41 (1.00-1.98)	**0.049**
rs3762993	5	151130672	Intron	119/159	94/176	C/T	0.428	0.348	0.344	1.40 (0.99-1.98)	0.055
rs9324677	5	151134177	Intron	114/164	111/159	A/C	0.41	0.411	0.86	1.00 (0.71-1.40)	0.98
rs13185706	5	151142998	Intron	35/243	39/231	C/A	0.126	0.144	1	0.85 (0.52-1.39)	0.525

SNP, single nucleotide polymorphism; MAF, minor allele frequency; HWE, Hardy-Weinberg equilibrium; OR, odds ratio; 95% CI, 95% confidence interval.

p values were calculated from χ^2^ test.

Bold values indicate statistical significance (p < 0.05).

As illustrated in **Table 3**, the results of this study demonstrated that the TC genotype of rs11960458was correlated with reduced risk of HNC compared with TT genotype (adjusted OR = 0.54, 95% CI = 0.31-0.94, *p* = 0.030). The CC+CT genotype of rs4958897 was found to be asociated with an increased HNC risk compared with the TT genotype (adjusted OR = 1.69, 95% CI = 1.03-2.78, *p* = 0.039).

**Table 3:** Association of *ANXA6* genetic variants and HNC susceptibility.

SNP-ID	Models	Genotypes	Cases	Controls	Without adjustment		With adjustment	
					OR (95% CI)	*p*	OR (95% CI)	*p*
rs11960458	Codominant	CC	51 (36.69%)	39 (28.89%)	1		1	
		TT	36 (35.90%)	23 (17.04%)	1.20 (0.61-2.34)	0.598	1.20 (0.61-2.34)	0.597
		TC	52 (37.41%)	73 (54.07%)	0.54 (0.31-0.94)	**0.03**	0.54 (0.32-0.94)	**0.03**
	Dominant	CC	51 (36.69%)	39 (28.89%)	1		1	
		TT+TC	88 (73.31%)	96 (71.11%)	0.70 (0.42-1.16)	0.17	0.70 (0.42-1.17)	0.17
	Recessive	TC+CC	103 (74.10%)	112 (82.96%)	1		1	
		TT	36 (35.90%)	23 (17.04%)	1.70 (0.95-3.06)	0.076	1.70 (0.95-3.07)	0.076
	Additive	—	/	/	1.02 (0.74-1.41)	0.904	1.02 (0.74-1.41)	0.903
rs4958892	Codominant	GG	64 (46.04%)	59 (43.70%)	1		1	
		AA	19 (13.67%)	23 (17.04%)	0.76 (0.38-1.54)	0.448	0.76 (0.38-1.54)	0.446
		AG	56 (40.29%)	53 (39.26%)	0.97 (0.58-1.63)	0.921	0.97 (0.58-1.63)	0.921
	Dominant	GG	64 (46.04%)	59 (43.70%)	1		1	
		AA+AG	75 (53.96%)	76 (56.30%)	0.91 (0.57-1.47)	0.697	0.91 (0.56-1.47)	0.697
	Recessive	AG+GG	120 (86.33%)	112 (82.96%)	1		1	
		AA	19 (13.67%)	23 (17.04%)	0.77 (0.40-1.49)	0.44	0.77 (0.40-1.49)	0.439
	Additive	—	/	/	0.90 (0.64-1.25)	0.511	0.89 (0.64-1.25)	0.51
rs78243462	Codominant	CC	119 (85.61%)	116 (85.93%)	1		1	
		TT	3 (2.16%)	1 (0.74%)	2.92 (0.30-28.52)	0.356	2.93 (0.30-28.65)	0.356
		TC	17 (12.23%)	18 (13.33%)	0.92 (0.45-1.87)	0.82	0.92 (0.45-1.88)	0.819
	Dominant	CC	119 (85.61%)	116 (85.93%)	1		1	
		TT+TC	20 (14.39%)	19 (14.07%)	1.03 (0.52-2.02)	0.941	1.03 (0.52-2.02)	0.943
	Recessive	TC+CC	136 (97.84%)	134 (99.26%)	1		1	
		TT	3 (2.16%)	1 (0.74%)	2.96 (0.30-28.78)	0.351	2.96 (0.30-28.92)	0.351
	Additive	—	/	/	1.11 (0.62-2.01)	0.722	1.11 (0.62-2.01)	0.723
rs4346760	Codominant	AA	29 (20.86%)	42 (31.11%)	1		1	
		CC	40 (28.78%)	32 (23.70%)	1.81 (0.93-3.51)	0.079	1.82 (0.93-3.54)	0.079
		CA	70 (50.36%)	61 (45.19%)	1.66 (0.93-2.98)	0.089	1.66 (0.93-2.99)	0.088
	Dominant	AA	29 (20.86%)	42 (31.11%)	1		1	
		CC+CA	110 (79.14%)	93 (66.91%)	1.71 (0.99-2.96)	0.054	1.72 (0.99-2.97)	0.054
	Recessive	CA+AA	99 (71.22%)	103 (76.30%)	1		1	
		CC	40 (28.78%)	32 (23.70%)	1.30 (0.76-2.23)	0.341	1.31 (0.76-2.25)	0.336
	Additive	—	/	/	1.34 (0.96-1.87)	0.08	1.35 (0.97-1.88)	0.079
rs4958897	Codominant	TT	42 (30.22%)	57 (42.22%)	1		1	
		CC	30 (21.58%)	23 (17.04%)	1.77 (0.90-3.47)	0.097	1.77 (0.90-3.47)	0.098
		CT	67 (48.20%)	55 (40.74%)	1.65 (0.97-2.82)	0.065	1.66 (0.97-2.84)	0.065
	Dominant	TT	42 (30.22%)	57 (42.22%)	1		1	
		CC+CT	97 (69.78%)	78 (57.78%)	1.69 (1.03-2.78)	**0.039**	1.69 (1.03-2.78)	**0.039**
	Recessive	CT+TT	109 (78.42%)	112 (82.96%)	1		1	
		CC	30 (21.58%)	23 (17.04%)	1.34 (0.73-2.45)	0.342	1.34 (0.73-2.46)	0.341
	Additive	—	/	/	1.37 (0.99-1.91)	0.06	1.37 (0.99-1.91)	0.06
rs3762993	Codominant	TT	46 (33.09%)	60 (44.44%)	1		1	
		CC	26 (18.71%)	19 (14.07%)	1.79 (0.88-3.61)	0.107	1.79 (0.88-3.64)	0.106
		CT	67 (48.20%)	56 (41.48%)	1.56 (0.93-2.63)	0.095	1.56 (0.93-2.64)	0.094
	Dominant	TT	46 (33.09%)	60 (44.44%)	1		1	
		CC+CT	93 (66.91%)	75 (55.56%)	1.62 (0.99-2.64)	0.054	1.62 (0.99-2.65)	0.054
	Recessive	CT+TT	113 (81.29%)	116 (85.93%)	1		1	
		CC	26 (18.71%)	19 (14.07%)	1.41 (0.74-2.68)	0.302	1.41 (0.74-2.69)	0.301
	Additive	—	/	/	1.38 (0.98-1.94)	0.063	1.38 (0.98-1.94)	0.062
rs9324677	Codominant	CC	50 (35.97%)	46 (34.07%)	1		1	
		AA	25 (17.995)	22 (16.30%)	1.05 (0.52-2.10)	0.901	1.05 (0.52-2.11)	0.898
		AC	64 (46.04%)	67 (49.63%)	0.88 (0.52-1.49)	0.631	0.88 (0.52-1.49)	0.633
	Dominant	CC	50 (35.97%)	46 (34.07%)	1		1	
		AA+AC	89 (64.03%)	89 (65.93%)	0.92 (0.56-1.51)	0.742	0.92 (0.56-1.51)	0.744
	Recessive	AC+CC	114 (82.01%)	113 (83.70%)	1		1	
		AA	25 (17.995)	22 (16.30%)	1.13 (0.60-2.11)	0.711	1.13 (0.60-2.12)	0.708
	Additive	—	/	/	1.00 (0.71-1.40)	0.98	1.00 (0.71-1.40)	0.983
rs13185706	Codominant	AA	108 (77.70%)	99 (73.33%)	1		1	
		CC	4 (2.88%)	3 (2.22%)	1.22 (0.27-5.60)	0.796	1.22 (0.27-5.60)	0.796
		CA	27 (19.42%)	33 (24.44%)	0.75 (0.42-1.34)	0.329	0.75 (0.42-1.34)	0.328
	Dominant	AA	108 (77.70%)	99 (73.33%)	1		1	
		CC+CA	31 (22.30%)	36 (26.67%)	0.79 (0.45-1.37)	0.401	0.79 (0.45-1.37)	0.401
	Recessive	CA+AA	135 (97.12%)	132 (97.78%)	1		1	
		CC	4 (2.88%)	3 (2.22%)	1.30 (0.29-5.94)	0.732	1.31 (0.29-5.95)	0.731
	Additive	—	/	/	0.86 (0.53-1.39)	0.538	0.86 (0.53-1.39)	0.538

SNP, single nucleotide polymorphism; OR, odds ratio; 95% CI, 95% confidence interval.

p^a^ values were calculated by logistic regression analysis with the comparison between diabetes patients and healthy controls.

p^b^ values were calculated by logistic regression analysis with adjustment for age and gender.

Bold values indicate statistical significance (p < 0.05).

To further investigate the associations of *ANXA6* SNPs with HNC risk, stratified analyses based on age, gender, and tumor sites were conducted. The results of age-stratification analysisshowed that rs4958897 was associated with an increased risk of HNC in individuals aged ≤ 53 years (CT vs. TT: OR = 2.64, 95% CI = 1.18-5.90, *p* = 0.018; CC+CT vs. TT: OR = 2.18, 95% CI = 1.04-4.56, *p* = 0.039), as shown in **Table 4**. The results of gender-stratification analysis indicated that the TC genotype of rs11960458 (TC vs. CC: OR = 0.50, 95% CI = 0.26-0.97, *p* = 0.040) and the CA genotype of rs13185706 (CA vs. CC: OR = 0.48, 95% CI = 0.24-0.96, *p* = 0.043) were associated with reduced HNC risk in males. However, rs4346760 was a risk factor for HNC in males (C vs. A: OR = 1.55, 95% CI = 1.04-2.31, *p* = 0.032; homozygous: OR = 2.31, 95% CI = 1.04-5.13, *p* = 0.039; heterozygous: OR = 2.17, 95% CI = 1.08-4.38, *p* = 0.030; additive: OR = 1.53, 95% CI = 1.02-2.27, *p* = 0.038), as shown in **Table 4**.

**Table 4:** Correlation of *ANXA6* variants with HNC risk stratified by age and gender.

SNP-ID	Models	Genotypes	Age > 53		Age ≤ 53		Males		Females	
			OR (95% CI)	*p*	OR (95% CI)	*p*	OR (95% CI)	*p*	OR (95% CI)	*p*
rs11960458	Allele	C	1		1		1		1	
		T	0.97 (0.61-1.55)	0.905	1.08 (0.66-1.76)	0.768	0.99 (0.66-1.48)	0.948	1.11 (0.60-2.06)	0.74
	Codominant	CC	1		1		1		1	
		TT	1.10 (0.44-2.77)	0.843	1.33 (0.50-3.58)	0.568	1.14 (0.51-2.54)	0.746	1.34 (0.39-4.58)	0.636
		TC	0.60 (0.28-1.27)	0.18	0.47 (0.21-1.05)	0.065	0.50 (0.26-0.97)	**0.04**	0.65 (0.24-1.79)	0.408
	Dominant	CC	1		1		1		1	
		TT+TC	0.73 (0.36-1.46)	0.367	0.66 (0.31-1.39)	0.27	0.65 (0.36-1.20)	0.169	0.82 (0.32-2.11)	0.687
	Recessive	TC+CC	1		1		1		1	
		TT	1.47 (0.65-3.33)	0.359	2.09 (0.88-4.95)	0.093	1.69 (0.84-3.42)	0.143	1.73 (0.59-5.05)	0.316
	Additive	—	0.98 (0.63-1.54)	0.94	1.07 (0.66-1.71)	0.789	0.99 (0.67-1.46)	0.95	1.10 (0.61-2.01)	0.75
rs4346760	Allele	A	1		1		1		1	
		C	1.25 (0.79-1.99)	0.344	0.68 (0.41-1.10)	0.116	1.55 (1.04-2.31)	**0.032**	1.00 (0.54-1.85)	0.997
	Codominant	AA	1		1		1		1	
		CC	1.58 (0.61-4.09)	0.343	0.43 (0.16-1.16)	0.095	2.31 (1.04-5.13)	**0.039**	1.01 (0.29-3.50)	0.987
		CA	1.11 (0.50-2.43)	0.804	1.11 (0.50-2.48)	0.802	2.17 (1.08-4.38)	**0.030**	0.87 (0.30-2.48)	0.789
	Dominant	AA	1		1		1		1	
		CC+CA	1.24 (0.60-2.58)	0.564	0.81 (0.39-1.71)	0.584	2.22 (1.15-4.28)	**0.017**	0.91 (0.34-2.46)	0.852
	Recessive	CA+AA	1		1		1		1	
		CC	1.49 (0.65-3.41)	0.342	0.41 (0.17-0.97)	0.043	1.41 (0.73-2.71)	0.302	1.11 (0.40-3.11)	0.841
	Additive	—	1.25 (0.78-1.99)	0.362	0.69 (0.43-1.12)	0.132	1.53 (1.02-2.27)	**0.038**	1.00 (0.54-1.86)	0.997
rs4958897	Allele	T	1		1		1		1	
		C	1.45 (0.91-2.33)	0.12	1.36 (0.83-2.23)	0.225	1.25 (0.8-1.88)	0.279	1.87 (0.99-3.53)	0.052
	Codominant	TT	1		1		1		1	
		CC	2.13 (0.82-5.50)	0.119	1.45 (0.54-3.88)	0.463	1.41 (0.63-3.18)	0.403	2.90 (0.85-9.94)	0.09
		CT	1.14 (0.54-2.38)	0.735	2.64 (1.18-5.90)	**0.018**	1.56 (0.82-2.94)	0.174	1.90 (0.70-5.20)	0.211
	Dominant	TT	1		1		1		1	
		CC+CT	1.37 (0.69-2.73)	0.363	2.18 (1.04-4.56)	**0.039**	1.51 (0.83-2.74)	0.173	2.20 (0.88-5.50)	0.093
	Recessive	CT+TT	1		1		1		1	
		CC	1.99 (0.84-4.71)	0.118	0.86 (0.36-2.09)	0.744	1.10 (0.53-2.28)	0.793	2.10 (0.69-6.42)	0.193
	Additive	—	1.40 (0.89-2.22)	0.148	1.34 (0.83-2.18)	0.231	1.24 (0.83-1.84)	0.292	1.73 (0.95-3.16)	0.075
rs13185706	Allele	A	1		1		1		1	
		C	0.52 (0.25-1.08)	0.075	1.35 (0.67-2.71)	0.396	0.74 (0.42-1.33)	0.316	1.22 (0.48-3.13)	0.675
	Codominant	AA	1		1		1		1	
		CC	/	/	4.28 (0.45-40.42)	0.205	3.53 (0.38-32.66)	0.267	/	/
		CA	0.57 (0.25-1.29)	0.181	0.94 (0.40-2.20)	0.891	0.48 (0.24-0.96)	**0.039**	2.72 (0.80-9.26)	0.109
	Dominant	AA	1		1		1		1	
		CC+CA	0.52 (0.23-1.15)	0.106	1.16 (0.52-2.56)	0.719	0.58 (0.30-1.13)	0.108	1.78 (0.59-5.33)	0.306
	Recessive	CA+AA	1		1		1		1	
		CC	/	/	4.33 (0.46-40.64)	0.2	4.10 (0.45-37.79)	0.213	/	/
	Additive	—	0.50 (0.24-1.07)	0.073	1.30 (0.67-2.51)	0.433	0.75 (0.43-1.33)	0.33	1.21 (0.48-3.01)	0.688

SNP, single nucleotide polymorphism; OR, odds ratio; 95% CI, 95% confidence interval.

p values were calculated by logistic regression analysis with adjustment for age and gender.

Bold values indicate statistical significance (p < 0.05).

Furthermore, the results of tumor sites stratification analysis obsevered that rs4346760 was correlated with an increased risk of nasopharyngeal carcinoma (NPC) under the allele (OR = 1.55, 95% CI = 1.04-2.31, *p* = 0.032), homozygous (OR = 2.35, 95% CI = 1.01-5.46, *p* = 0.047), heterozygous (OR = 2.43, 95% CI = 1.14-5.18, *p* = 0.022), and dominant models (OR = 2.40, 95% CI = 1.17-4.93, *p* = 0.017). Moreover, rs4958897 (C vs. T: OR = 1.55, 95% CI = 1.04-2.31, *p* = 0.032; CC+CT vs. TT: OR = 1.93, 95% CI = 1.05-3.55, *p* = 0.035; additive: OR = 1.51, 95% CI = 1.02-2.24, *p* = 0.039) and rs3762993 (C vs. T: OR = 1.52, 95% CI = 1.01-2.28, *p* = 0.042; CC+CT vs. TT: OR = 1.93, 95% CI = 1.06-3.51, *p* = 0.033; additive: OR = 1.52, 95% CI = 1.01-2.28, *p* = 0.041) were also found to be associated with increased risk of NPC, as presented in **Table 5**.

**Table 5:** Association of *ANXA6* polymorphisms and HNC risk stratified by tumor sites.

SNP-ID	Models	Genotypes	Nasopharynx				Larynx			
			Cases	Controls	OR (95% CI)	*p*	Cases	Controls	OR (95% CI)	*p*
rs4346760	Allele	A	66	145	1		47	145	1	
		C	88	125	1.55 (1.04-2.31)	**0.032**	39	125	0.96 (0.59-1.57)	0.878
	Codominant	AA	12	42	1		14	42	1	
		CC	23	32	2.35 (1.01-5.46)	**0.047**	10	32	1.12 (0.42-3.00)	0.821
		CA	42	61	2.43 (1.14-5.18)	**0.022**	19	61	0.97 (0.41-2.27)	0.94
	Dominant	AA	12	42	1		14	42	1	
		CC+CA	65	93	2.40 (1.17-4.93)	**0.017**	39	91	1.02 (0.47-2.22)	0.961
	Recessive	CA+AA	54	103	1		33	103	1	
		CC	23	32	1.28 (0.68-2.43)	0.448	10	32	1.14 (0.48-2.72)	0.766
	Additive	—			1.49 (0.99-2.22)	0.054	/	**/**	1.05 (0.64-1.72)	0.843
rs4958897	Allele	T	85	176	1		53	176	1	
		C	74	101	1.55 (1.04-2.31)	**0.032**	33	94	1.33 (0.81-2.17)	0.262
	Codominant	TT	21	57	1		17	60	1	
		CC	18	23	2.19 (0.98-4.87)	0.056	7	19	1.70 (0.60-4.83)	0.32
		CT	38	55	1.82 (0.95-3.51)	0.073	19	56	1.39 (0.61-3.16)	0.439
	Dominant	TT	21	57	1		17	60	1	
		CC+CT	56	78	1.93 (1.05-3.55)	**0.035**	26	75	1.47 (0.68-3.17)	0.327
	Recessive	CT+TT	59	112	1		36	116	1	
		CC	18	23	1.56 (0.77-3.15)	0.213	7	19	1.42 (0.56 -3.62)	0.461
	Additive	—	/	/	1.51 (1.02-2.24)	**0.039**	/	/	1.32 (0.79-2.19)	0.292
rs3762993	Allele	T	80	169	1		48	169	1	
		C	69	94	1.52 (1.01-2.28)	**0.042**	38	101	1.17 (0.71-1.93)	0.549
	Codominant	TT	23	60	1		14	57	1	
		CC	15	19	2.14 (0.92-4.96)	0.076	9	23	1.47 (0.49-4.39)	0.495
		CT	39	56	1.85 (0.98-3.51)	0.057	20	55	1.00 (0.45-2.24)	0.998
	Dominant	TT	23	60	1		14	57	1	
		CC+CT	54	75	1.93 (1.06-3.51)	**0.033**	29	78	1.10 (0.52-2.33)	0.8
	Recessive	CT+TT	62	116	1		34	112	1	
		CC	15	19	1.52 (0.71-3.21)	0.279	9	23	1.47 (0.53-4.06)	0.462
	Additive	—	/	/	1.52 (1.02-2.28)	**0.041**	/	/	1.16 (0.68-1.96)	0.584

SNP, single nucleotide polymorphism; OR, odds ratio; 95% CI, 95% confidence interval.

p values were calculated by logistic regression analysis with adjustment for age and gender.

Bold values indicate statistical significance (p < 0.05).

**Figure 1:**
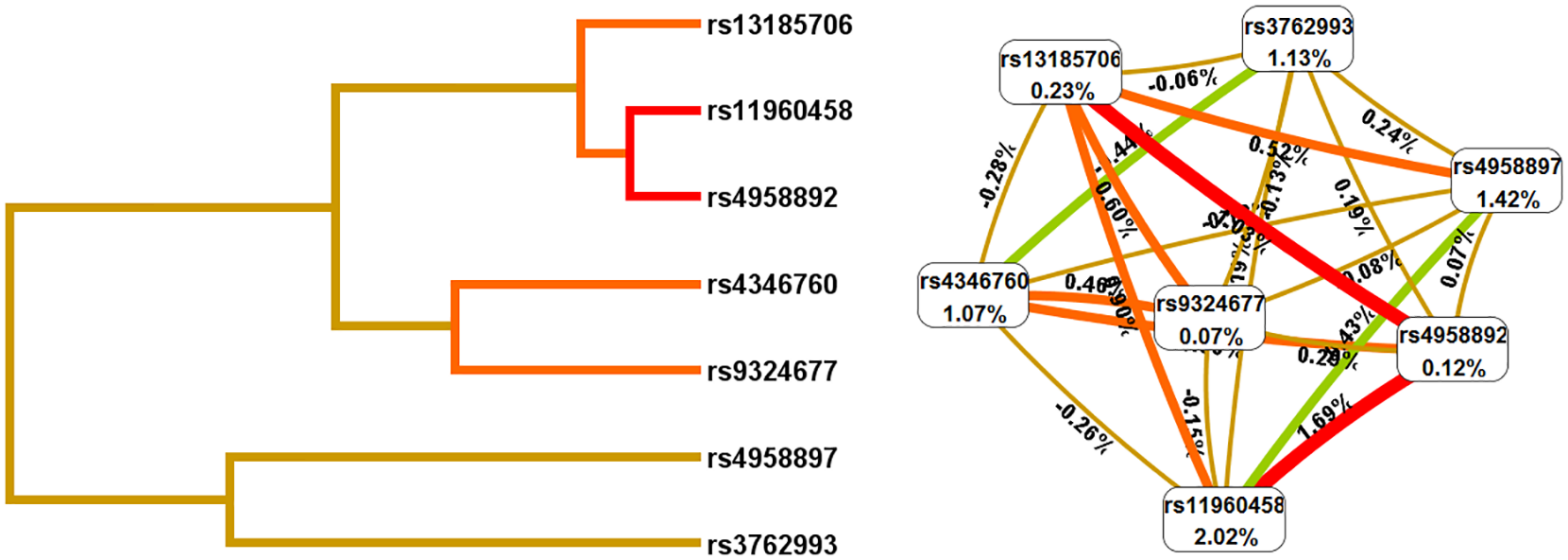
The SNP-SNP interaction analysis (Dendrogram and Fruchterman-Reingold). The colors represent synergy or redundancy. Green and blue with a negative correlation; red, orange and brown with a positive correlation.).

In addition, we used the MDR method to analyze the SNP-SNP interactions (**Figure 1** and **Table 6**). These results revealed that rs11960458 and rs4958892 had a positive synergistic effect on increased HNC risk. However, rs11960458 and rs4958897 had a negative synergistic effect on HNC risk. The two-locus model (rs11960458 and rs4958892) had the highest Cross-validation (CV) consistency and balanced accuracy (Bal. Acc) testing. (CV Consistency: 9/10; Testing Bal. Acc.: 0.596).

**Table 6:** The SNP-SNP interactions analysis.

Model	Bal.Acc.CV Training	Bal.Acc.CV Testing	CV Consistency
rs11960458	0.583	0.537	8/10
rs11960458,rs4958892	0.615	0.596	9/10
rs11960458,rs4958892,rs4958897	0.661	0.556	8/10
rs11960458,rs4958892,rs4346760,rs4958897	0.711	0.522	7/10
rs11960458,rs4958892,rs3762993,rs9324677,rs13185706	0.772	0.526	7/10
rs11960458,rs4958892,rs4346760,rs3762993,rs9324677,rs13185706	0.821	0.456	8/10

Bal. Acc., Balanced accuracy; CVC, Cross-validation consistency.

## Discussion

This case-control study observed that rs4958897 was associated with an increased risk of HNC, while rs11960458 was linked to a reduced risk of HNC. Age and gender stratification results revealed that *ANXA6* polymorphisms (rs11960458, rs4958897, rs4346760, and rs13185706) were significantly related to the susceptibility to HNC. Furthermore, rs4346760, rs4958897, and rs3762993 were found to be associated with the risk of nasopharyngeal carcinoma. These results highlighted the importance of the *ANXA6* gene in the occurrence and development of HNC, and confirmed that *ANXA6* might be a potential target for HNC prognosis and diagnosis.

Annexin is a calcium-dependent superfamily of proteins that can bind negatively charged membrane phospholipids and is a highly abundant protein. Annexin has been studied in laryngeal carcinoma, nasopharyngeal carcinoma and other head and neck tumors. For example, Luo et al. have uncovered that *ANXA2* is highly expressed in laryngeal carcinoma and its expression is associated with tumor size, distant metastasis and clinical stage (24). Others have also illustrated that *ANXA1* and *ANXA2* could facilitate the progression of NPC (25, 26). As far as we know, the sequences of *ANXA6* are highly similar to those of *ANXA1* and *ANXA2*. *ANXA6*, a member of annexin superfamily, is located on human chromosome 5q33.1 and contains 26 exons with a length of about 60kbp. Some literatures have demonstrated that *ANXA6* is involved in cell growth, differentiation, invasion, and motility in many cancers (27, 28). Furthermore, Chen et al. have observed that *ANXA6* promotes autophagy through suppressing the PI3K/AKT/mTOR pathway, thereby upregulating radioresistance in NPC (29). These reports suggest that *ANXA6* may play an important role in HNC and other malignant tumors. Nevertheless, there are few studies on the role of *ANXA6* in HNC development at present.

In this study, the linkage between *ANXA6* SNPs and HNC risk in the Chinese people was assessed. Overall analysis results indicated that the C allele and CC+CT genotypes of rs4958897 were associated with increased risk of HNC. However, individuals with the TC genotype of rs11960458 had lower risk of HNC compared with those with the TT genotype Rs11960458 is located in the 3’-UTR region of miRNA-binding site of the *ANXA6* gene. Therefore, we speculated that rs11960458 affected the expression of *ANXA6* and had a protective effect on HNC by maintaining mRNA stability and miRNA binding activity. However, our hypothesis requires functional studies to confirm.

Age stratification results showed that rs4958897 was a risk factor for HNC in aged ≤ 53. Furthermore, the TC genotype of rs11960458 and CA genotype of rs13185706 were found to be associated with reduced HNC risk, while rs4346760 was related to increased risk of HNC in males. Three *ANXA6* SNPs (rs4346760, rs4958897, and rs3762993) facilitated the occurrence of nasopharyngeal carcinoma. However, no association between eight SNPs in *ANXA6* and risk of HNC was found in subgroups of those aged > 53, female, and with laryngeal carcinoma. These findings suggested that genetic susceptibility to HNC varied by age, gender and types of HNC. An epidemiological study indicated that the incidence of HNC differed among people of different sexes and ages, and is higher in males and the elderly (30). Males are much more susceptible to HNC than females, and this difference is mainly due to the discrepancies in the lower part of the upper aerodigestive tract, such as larynx and hypopharynx (31). Therefore, the importance of heterogeneity should be considered in the genetic association study of HNC risk.

In addition, SNP-SNP interaction results showed that rs11960458, rs4958892, rs4346760, and rs3762993 had positive synergistic effect on increased HNC risk. However, rs11960458 and rs4958897 had negative synergistic effect on HNC risk. These four SNPs (rs4346760, rs4958897, rs3762993, and rs13185706) are located in the intron region of the *ANXA6* gene. Combining previous studies and database predictions, we hypothesized that *AXAN6* intron SNPs could lead tochanges in *ANXA6* expression and activity *via* influencing mRNA splicing, and ultimately affecting disease susceptibility. Further studies are needed to explore the specific role of these *ANXA6* SNPs.

Although the association of *ANXA6* with HNC susceptibility was detected in this study, there are still some limitations. Firstly, there are no supporting studies about these SNPs, but the good thing is this study isfirst to report the association between eight *ANXA6* SNPs (rs11960458, rs4958892, rs78243462, rs4346760, rs4958897, rs3762993, rs9324677, and rs13185706) and risk of HNC in the Chinese Han population. Secondly, the subjects in this study were recruited from the same hospital, so there were geographic limitations on sample selection. Therefore, further studies with large samples are needed to validate our findings of *ANXA6* as a biomarker for HNC.

## Conclusions

In conclusion, these results demonstrate that polymorphisms (rs11960458, rs4346760, rs495889, rs3762993 and rs13185706) in the *ANXA6* gene are related to the susceptibility to HNC in the Chinese Han population, indicating that *ANXA6* may serve as a diagnostic and prognostic molecular biomarker for patients with HNC.

## Data availability statement

The original contributions presented in the study are included in the article/supplementary material. Further inquiries can be directed to the corresponding author.

## Ethics statement

The studies involving human participants were reviewed and approved by Ethics Committee of People’s Hospital of Wanning (No. SL-2023-001). The patients/participants provided their written informed consent to participate in this study.

## Author contributions

WX: drafted and revised important content. ZL and XZ: performed experiments. JC, ZC and XY: analyzed data. YD: conceived and designed experiments. All authors contributed to the article and approved the submitted version.
